# The sexual spore pigment asperthecin is required for normal ascospore production and protection from UV light in *Aspergillus nidulans*

**DOI:** 10.1093/jimb/kuab055

**Published:** 2021-12-23

**Authors:** Jonathan M. Palmer, Philipp Wiemann, Claudio Greco, Yi Ming Chiang, Clay C. C. Wang, Daniel L. Lindner, Nancy P. Keller

**Affiliations:** 1 Department of Medical Microbiology & Immunology, University of Wisconsin–Madison, Madison, WI 53706, USA; 2 Departments of Chemistry and Pharmacology & Pharmaceutical Sciences, University of Southern California, Los Angeles, CA 90089, USA; 3 Department of Bacteriology, University of Wisconsin–Madison, Madison, WI 53706, USA; 4 Northern Research Station, USDA Forest Service, Madison, WI 53726, USA

**Keywords:** Polyketide, UV protection, Ascospore, Cleistothecia, Fungi

## Abstract

Many fungi develop both asexual and sexual spores that serve as propagules for dissemination and/or recombination of genetic traits. Asexual spores are often heavily pigmented and this pigmentation provides protection from UV light. However, little is known about any purpose pigmentation that may serve for sexual spores. The model Ascomycete *Aspergillus nidulans* produces both green pigmented asexual spores (conidia) and red pigmented sexual spores (ascospores). Here we find that the previously characterized red pigment, asperthecin, is the *A. nidulans* ascospore pigment. The asperthecin biosynthetic gene cluster is composed of three genes: *aptA, aptB,* and *aptC,* where deletion of either *aptA* (encoding a polyketide synthase) or *aptB* (encoding a thioesterase) yields small, mishappen hyaline ascospores; while deletion of *aptC* (encoding a monooxygenase) yields morphologically normal but purple ascospores. Δ*aptA and* Δ*aptB* but not Δ*aptC* or wild type ascospores are extremely sensitive to UV light. We find that two historical ascospore color mutants, *clA6* and *clB1,* possess mutations in *aptA* and *aptB* sequences, respectively.

## Introduction

Sporulation is an essential developmental program for most fungi. A common characteristic of fungi, particularly members of the taxon Ascomycota, is the differentiation in time and space of asexual (mitotically derived) and sexual (meiotically derived) spores (Han et al., 2020; Zhang et al., 2020). Termed conidia and ascospores respectively, these two spore types are usually associated with long distance spread (conidia) or recombination in the sexual fruiting body, the ascocarp. Ascospores may or may not be airborne depending on the mechanism of ascocarp release. All *Aspergillus* spp. with a known sexual stage bear ascospores in enclosed ascocarps with dissolution of ascocarp cell walls/membranes leading to ascospore release.

Both spore types can be pigmented or hyaline (lacking pigmentation). Typically, airborne conidia are heavily pigmented, a finding that led to early speculation that pigments protected the conidia from UV radiation. This hypothesis has been largely borne out in studies of numerous fungi where pigment deficient conidia are more sensitive to UV radiation. Examples include *Aspergillus fumigatus* (Blachowicz et al., 2020)*, Aspergillus niger* (Esbelin et al., 2013), *Cochliobolus (Bipolaris*) *heterostrophus* (Leonard, 1977), *Ashbya gossypii* (Stahmann et al., 2001), *Alternaria brassicicola* (Cho et al., 2012), *Ustilago violacea* (Will et al., 1984), and *Neurospora crassa* (Blanc et al., 1976). In contrast, while some ascospores are airborne, particularly those produced by a subset of plant pathogenic Ascomycetes (Churchill, 2011; Gimeno et al., 2020), most ascospores are not commonly found in the air but formed in the organic debris of soils where ascocarps serve as resistant fungal tissues favorable for overwintering or surviving other harsh environmental conditions (Jeger, 1984; Mmbaga, 2000).

Although ascospores can be pigmented, few studies have examined either the genetic pathway of ascospore pigmentation or any role of pigments in sexual spore survival. The ascospores of the genetic model *Aspergillus nidulans* are deep red in color. One of the first studies to address the chemistry of ascospore pigmentation in this fungus found that the pigment was a polyketide-derived dimeric hydroxylated anthraquinone (Brown & Salvo, 1994). Genetic studies of *A. nidulans* identified both colorless (*cl*) and blue (*bl*) variants of the red ascospore color (Apirion, 1963; Peintner & Rainer, 1999). The genes in the pigmentation pathway of conidia in *A. nidulans* are not physically linked (Mayorga & Timberlake, 1990; O’Hara & Timberlake, 1989), unlike the melanin gene cluster of the grey/blue polyketide *A. fumigatus* conidial pigment (Tsai et al., 1999) and most other fungal specialized metabolic pathways (Kjaerbolling et al., 2019). Therefore we investigated if the polyketide pathway of the anthraquinone red ascospore pigment would be arranged in a gene cluster and contain genes representing the *cl* and *bl* mutants of previous genetic studies. We present our findings that the ascospore pigment of *A. nidulans* is formed by the previously characterized three gene asperthecin biosynthetic gene cluster (BGC) and that two historical *cl* mutants represent mutants in the polyketide synthase AptA and the metallo-*β*-lactamase-type thioesterase AptB. Loss of AptA or AptB yields misshapen, small ascospores highly sensitive to UV radiation.

## Materials and Methods

### Fungal Strains

Fungal strains are listed in Table 1 and primers are listed in Supplementary Table S1. All fungal strains were maintained on glucose minimal media (GMM) and, when appropriate, medium was supplemented for growth of auxotrophic strains according to standard protocols. Creation of isogenic asperthecin mutant strains was done by crossing RDIT55.37 (*pyroA4, veA*+*)* to each of the single gene deletion strains of the asperthecin cluster (LO2131–Δ*aptA*, LO2435–Δ*aptB*, and LO2440–Δ*aptC*) (Szewczyk et al., 2008). The single Δ*aygA* mutant was obtained by deletion of *aygA* using *pyroA* as a marker. The double deletion strain Δ*aptC*/Δ*aygA* was created by deletion of *aygA* with *riboB.* The single *aygA* deletion mutant was confirmed by Southern analysis (Supplementary Fig. S1) while the double mutant was confirmed via diagnostic PCR (data not shown).

### Quantification of Ascospores

Sexual development was induced in *A. nidulans* by overlay inoculation of 1 × 10^6^ spores on Champe’s medium (Champe & el-Zayat, 1989) agar petri plates and subsequently incubated in the dark at 37°C for 7 days. Ascospores were quantified from sexual developmentally induced plates by homogenization of three 10-mm cores from each plate in 3 ml of sterile water and enumerated using a hemacytometer. If asci were present with visible immature ascospores inside, each ascus was counted as eight ascospores. Four biological replicates were completed for each strain and statistical significance was calculated with an ANOVA using Tukey multiple comparison posttest with Prism 6 (GraphPad).

### Sensitivity to DNA Damaging Agents

To obtain nearly pure ascospores, cleistothecia were harvested from sexually developmentally induced plates, broken open in sterile 15 ml round bottom polystyrene tubes containing 5 ml of water, filtered through sterile miracloth, and ascospores were enumerated using a hemacytometer. Approximately 75 ascospores were spread plate onto solid agar medium and exposed to UV-C (254 nm) irradiation using a UV crosslinker, incubated at 37°C for 24–48 h, and colony forming units (CFUs) were counted. All CFU data was normalized to percent survival by comparing CFUs on the treated plates to nontreated control plates for each strain. Four biological replicates were conducted for each fungal strain and statistical significance was calculated at the 50 mJ/cm^2^ treatment with an ANOVA using Tukey multiple comparison posttest with Prism 6 (GraphPad). Sensitivity to UV-induced DNA damage was compared to sensitivity to methyl methanesulfonate (MMS) by spot plating serial dilutions of ascospores onto GMM plates that were treated with UV light or onto GMM plates amended with different concentrations of MMS.

### Developmental mRNA Expression of Asperthecin Cluster

Wild-type *A. nidulans* (RJMP103.5) was overlay inoculated on Champe’s medium at a density of 1 × 10^6^ spores per petri dish. Asexually induced cultures were incubated at 37°C in constant light and sexually induced cultures were incubated at 37°C in constant darkness. Tissue was harvested at several time points by scraping mycelia/conidia/cleistothecia from the surface of petri dishes using a glass slide. Tissue was lyophilized overnight and total RNA was extracted using the TRIzol Plus RNA Purification Kit (TermoFisher #12183555) according to the manufacturer’s recommendations. Northern analysis was conducted and radiolabeled probes were generated from ~1 kb PCR products specific to each gene (Supplementary Table S2).

### Secondary Metabolites Extraction

Different strains were point inoculated (10^6^ spores) on Champe’s media and grown at 37°C in the dark for 18 days. Cleistothecia from sexually induced cultures were scrapped off the surface using a glass slide, transferred to a glass vial, homogenized, and extracted using acetonitrile/formic acid/dimethyl sulfoxide (98.5:0.5:1). The rest of the plate was blended and extracted with ethyl acetate (100 ml). After 2 h, the solid was removed using vacuum filtration and the organic layer was washed with water (3 × 50 ml). The combined organic phases were dried over anhydrous magnesium sulfate and concentrated under reduced pressure. The crude extracts were resuspended in LCMS grade acetonitrile to obtain a concentration of 10 mg/ml, filtered through a PTFE 0.2 *μ*m Teflon syringe filter.

### HPLC-DAD

HPLC-DAD for analysis was performed on Gilson GX-271 Liquid Handler with system 322 H2 Pump connected to a 171 Gilson Diode Array Detector and fraction collector. A XBridge BEH C18 XP Column (130 Å, 2.5 *μ*m, 4.6mm × 100mm) with XBridge BEH C18 XP VanGuard Cartridge (130 Å, 2.5 *μ*m, 3.9mm × 5mm) was used for analytical run with a flow rate of 0.8 ml/min. HPLC-grade water with 0.5% formic acid (solvent A) and HPLC-grade acetonitrile with 0.5% formic acid (solvent B) were used with the following gradients: 0min, 20% Solvent B; 2min, 20% Solvent B; 15 min, 95% Solvent B; 18 min, 95% Solvent B; 20 min, 20% Solvent B. Data acquisition and procession for the HPLC-DAD were controlled by TRILUTION LC V3.0.

### UHPLC-HRMS

UHPLC-HRMS was performed on a Thermo Scientific-Vanquish UHPLC system connected to a Thermo Scientific Q Exactive Orbitrap mass spectrometer in ES^+^ and ES^−^ mode between 200 m/z and 1000 m/z to identify metabolites. A Zorbax Eclipse XDB-C18 column (2.1×150mm, 1.8*μ*m particle size) was used with a flow rate of 0.2 ml/min for all samples. LCMS-grade water with 0.5% formic acid (solvent A) and LCMS-grade acetonitrile with 0.5% formic acid (solvent B) were used with the following gradients: 0min, 20% Solvent B; 2min, 20% Solvent B; 15 min, 95% Solvent B; 18 min, 95% Solvent B; 20min, 20% Solvent B. Nitrogen was used as the sheath gas. Data acquisition and procession for the UHPLCMS were controlled by Thermo Scientific Xcalibur software.

### Whole Genome Resequencing of Historical Ascospore Color Mutants

Three historical ascospore color mutants (*clA6*, *clB1*, and *blA1*) were obtained from the Fungal Genetics Stock Center (McCluskey et al., 2010) and each color mutant was subsequently crossed with RJMP139.13 to create single mutant strains in a similar genetic background (*pyroA4, veA*+). One isolate from each cross as well as a wild-type isolate obtained from one of the crosses (RJMP250.1, RJMP250.11, RJMP251.3, and RJMP252.14) were sequenced on the Ion Torrent Personal Genome Machine. High molecular weight DNA was isolated as described in Palmer et al. (2014) and DNA-sequencing libraries for each isolate were constructed using the Ion Xpress Plus Fragment Library Kit (#4471269), templated onto Ion Sphere Particles using the Ion PGM Template OT2 400 Kit (#4479878), and sequenced using the Ion PGM 400 bp Sequencing Kit (#4482002) using an Ion 318v2 (#4484355) chip according to manufacturer’s recommendations. Single nucleotide polymorphisms (SNPs) were identified against the *A. nidulans* FGSCA4 reference genome (obtained from www.aspgd.org) using CLC genomics Workbench 8.5.1 (Qiagen) as previously described (Pfannenstiel et al., 2017).

## Results

### Asperthecin is the Ascospore Pigment

The sexual spore (ascospore) pigment of *A. nidulans* has previously been chemically characterized as the polyketide ascoquinone A (Brown & Salvo, 1994); however, no secondary metabolite gene cluster has been linked to production of this compound. A forward genetics screen by Apirion (1963) identified three ascospore color mutants in *A. nidulans*: *clA6* produced clear ascospores, *clB1* also produced clear ascospores, and *blA1* produced blue ascospores. To determine which of the secondary metabolite gene clusters of *A. nidulans* was involved in ascospore pigmentation, we obtained strains harboring these mutations from the Fungal Genetics Stock Center (Table 1) and subsequently outcrossed each strain once to generate near isogenic backgrounds (*pyroA4, veA*+). Whole genome sequencing was employed for each mutant strain (RJMP251.3, RJMP252.15, RJMP250.11) as well as a near isogenic wild-type strain (RJMP250.1). Using SNP analysis from these data, we were able to identify mutations in the clear ascospore mutants, *clA6* and *clB1*. Both strains identified SNPs in the previously characterized three-gene cluster responsible for asperthecin production (Szewczyk et al., 2008). The *clA6* mutant (RJMP251.3) has a two base pair insertion in the coding region of PKS-encoding *aptA* that results in a frame-shift mutation at Leu-1151. In the *clB1* mutant, we identified a G→A mutation at position 902 in the coding region of the stand-alone thioesterase-encoding *aptB*, which resulted in mutating the Trp-301 to an early termination signal. We were unable to identify mutations in the *aptC* gene or surrounding genes in the blue ascospore mutant *blA1*. Through sexual crosses, the *blA1* mutation was mapped to the right arm of chromosome II (Apirion, 1963). We were unable to identify a specific causative mutation in this region; however, we did identify a large deletion near the right telomere of chromosome 2 from AN3500 to the end of the chromosome (~100 kB) containing ca 30 putative genes including the characterized fellutamide gene cluster (Yeh et al., 2016 and Table 2). The *apt* cluster is located on chromosome 3.

**Fig. 1:**
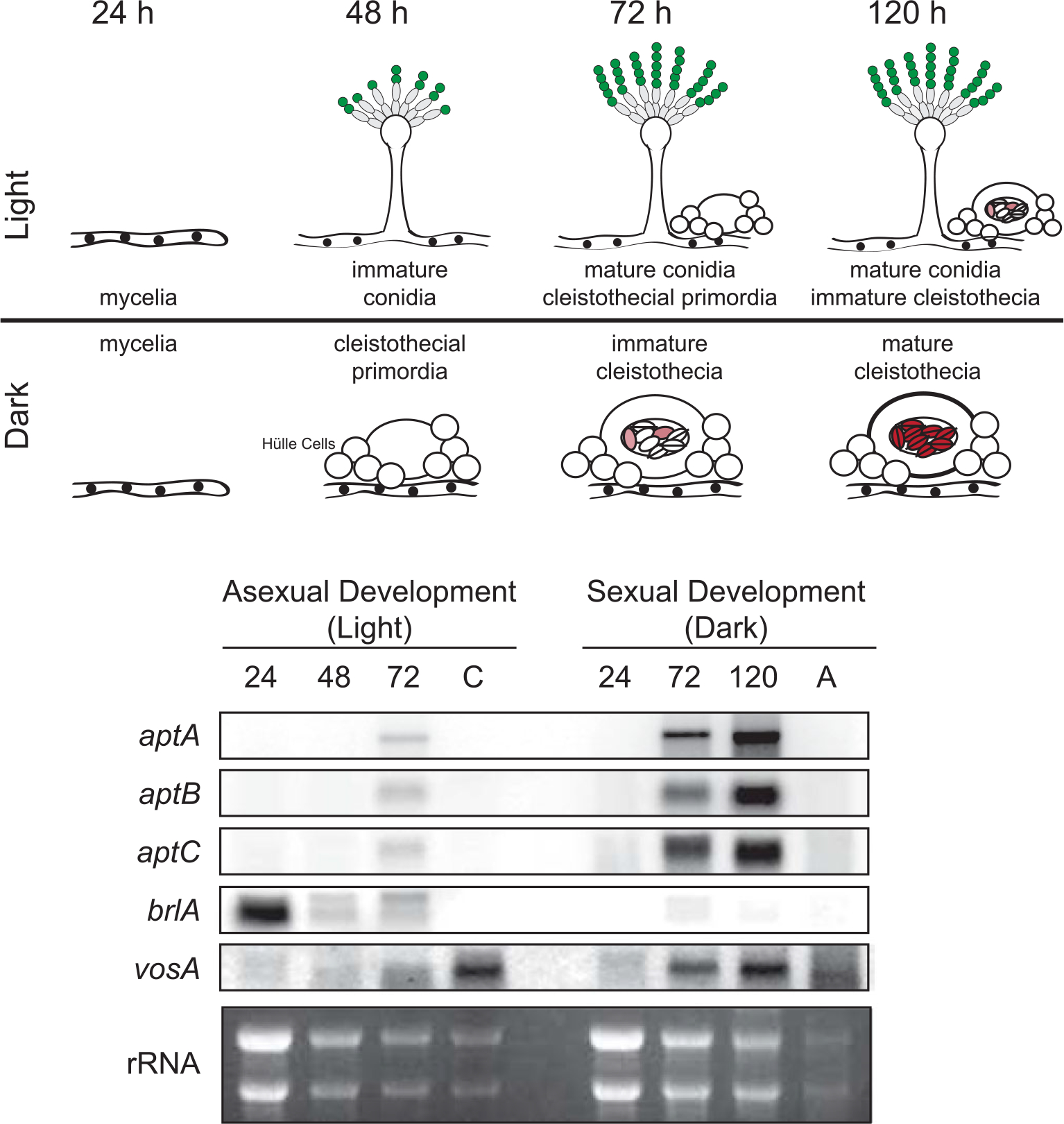
Asperthecin cluster genes are expressed during sexual development but they are not expressed in ascospores. (Top panel) *Aspergillus nidulans* development in light and dark regimes. Green conidia (mitotic spores) are produced on asexual conidiophores and red ascospores (meiotic spores) are produced with in the sexual fruiting body, the cleistothecium. (Bottom panel) mRNA extracted from light and dark regimes show that the three asperthecin genes (*aptA, aptB,* and *aptC*) are highly expressed during sexual development but not in ascospores. BrlA is a transcription factor required for conidiophore initiation and VosA is a regulatory protein required for maturation of both conidia and ascospores.

To determine if the asperthecin gene cluster was turned on during sexual development, we looked at expression levels during darkness (inducing sexual development) and in light (inducing asexual development) in a wild-type *A. nidulans* genetic background (Fig. 1). Using the asexual development regulator *brlA* and the spore specific *vosA* as controls to ensure proper developmental differentiation, we found that the asperthecin gene cluster (*aptA, aptB,* and *aptC*) is transcriptionally active during sexual development although not expressed in mature ascospores (Fig. 1).

**Fig. 2:**
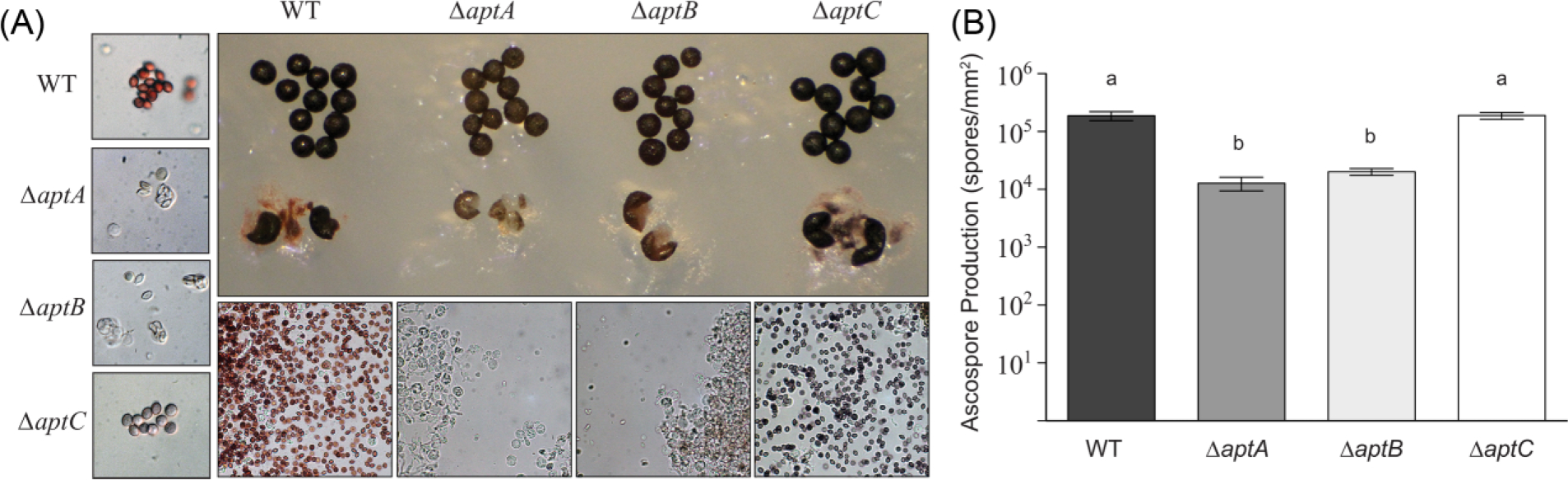
Asperthecin gene cluster deletion mutants have ascospore pigment and morphology phenotypes. (A) Deletion of the polyketide synthetase AptA or the beta-lactamase AptB results in hyaline and immature ascospore production. Cleistothecial cell walls are lighter in pigmentation compared to wild type and ascospores are commonly found contained in asci. Deletion of AptC, a putative monooxygenase, results in purple-blue ascospore pigmentation but wild-type ascospore and asci morphology. (B) Mutants with hyaline ascospore production are produced at an order of magnitude less than wild-type or Δ*aptC* mutants.

Szewczyk et al. (2008) characterized the asperthecin gene cluster in a Δ*sumO* deletion background, which renders the fungus very “sick” and unable to induce sexual development. We constructed new asperthecin gene cluster *(*Δ*aptA,* Δ*aptB,* Δ*aptC*) deletion mutants (Table 1) in a *veA*+ genetic background through sexual crosses. Morphological characterization of cleistothecia in these genetic backgrounds revealed that deletion of the polyketide synthase, PKS (*aptA*) or the metallo-*β*-lactamase-type thioesterase (*aptB*) resulted in clear (hyaline) ascospores, matching their forward genetic counterpart strains, *clA6* and *clB1.* Deletion of the flavin-dependent monooxygenase (*aptC*) resulted in ascospores that were visually blue-purple in color (Fig. 2A). Quantification of ascospores from these mutant strains also revealed that deletion of either *aptA* or *aptB* resulted in a reduction in the number of ascospores produced (Fig. 2B). Moreover, ascospores from these strains appeared to be immature as they were often found still inside asci even after prolonged incubation, and those ascospores that were released were misshapen (Fig. 2A). However, the blue-purple ascospores produced by Δ*aptC* strains appeared to be developmentally mature and were produced at wild-type levels (Fig. 2).

### Asperthecin and Pigmented Precursor Confer Resistance to UV Radiation

**Fig. 3:**
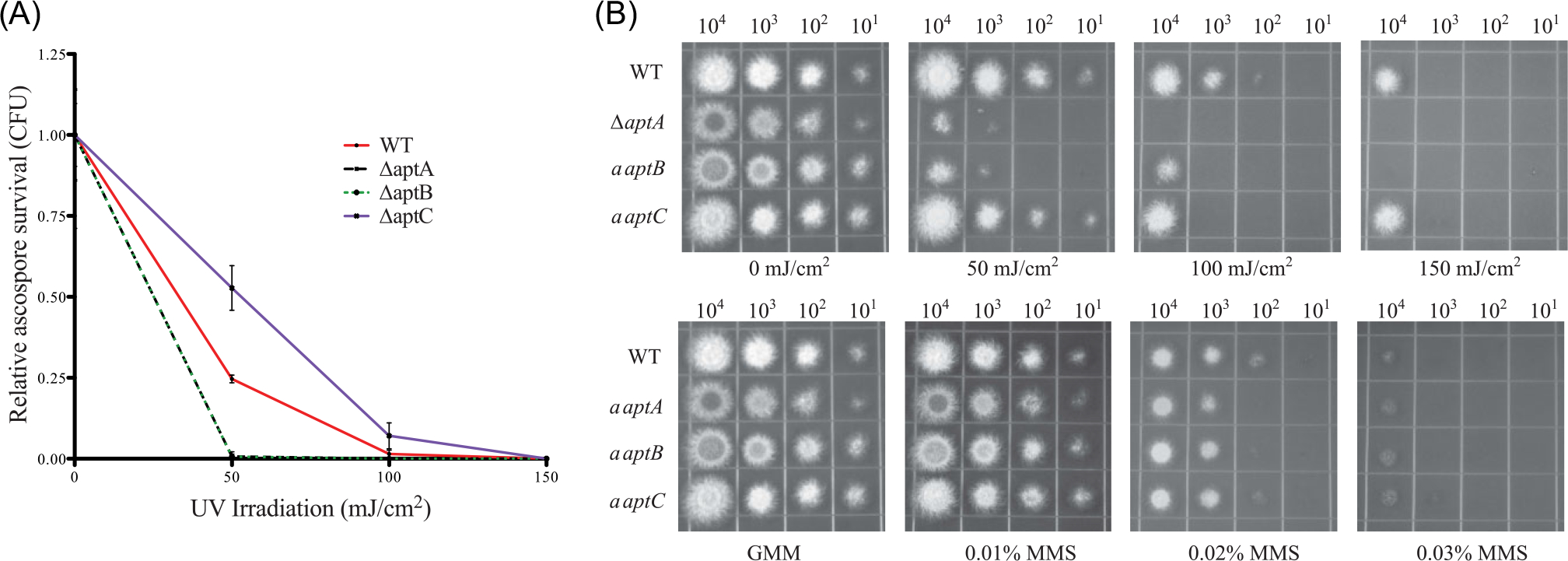
Asperthecin protects sexual spores from UV light. (A) Δ*aptC* ascospores are slightly more resistant than wild type to UV light, while both Δ*aptA* and Δ*aptB* ascospores are very sensitive to UV damage. Error bars represent standard error of four biological replicate experiments. Statistical significance was calculated using the 50 mJ/cm^2^ treatment with an ANOVA and Tukey multiple comparison posttest (*P* < 0.001). (B) Comparison of dilution plating of ascospores from wild type and *apt* mutants from UV and methyl methanesulfonate (MMS) (DNA damaging agent) treatment demonstrates that UV sensitivity is a result of loss of pigment protection and not of DNA repair machinery.

Many spore pigments are known to protect spores from UV radiation. Therefore we tested the ability of asperthecin to confer resistance to UV light. Ascospores were exposed to different exposures of UV-C (254 nm) light and survival was measured by taking a ratio of CFUs from UV treated plates versus untreated control plates. The clear ascospores from Δ*aptA* and Δ*aptB* displayed an increased sensitivity to UV light compared to wild-type red ascospores at 50 mJ/cm^2^ treatment (Fig. 3A, B). Interestingly, the blue-purple ascospores from the Δ*atpC* mutant were slightly more resistant to UV light than the wild type. UV light induces pyrimidine-dimer damage in DNA and must be repaired in order for the fungus to survive, thus the UV light survival assay is measuring two distinct “resistance” pathways, the first being the ability of the spore metabolites (pigments) to absorb UV light thereby protecting the DNA from damage, while the survival assay is also measuring the ability of the fungus to repair damaged DNA. These two pathways can be distinguished by measuring sensitivity to MMS, a chemical mimic of pyrimidine-dimer damage. Fungal mutants that are differentially sensitive to both UV light and MMS damage would be indicative of a defect in DNA repair pathway, whereas sensitivity to only UV light and not MMS is indicative of a fungal mutant that is defective in “physical” protection, that is, pigments in the spore. None of the asperthecin gene cluster mutants were susceptible to MMS in an ascospores spot dilution plate assay (Fig. 3B), thus supporting a role of physical protection for asperthecin.

### Apt Enzymes are Expressed in Developing Cleistothecia

Based on gene expression analysis, the asperthecin genes *aptA*, *aptB*, and *aptC* were specifically turned on during cleistothecial maturation of sexual development (Fig. 1). Moreover, these data showed that transcripts from all three enzymes are absent from ascospores as well as from asexual developmental tissue (Fig. 1), suggesting that the biosynthetic cluster is active during ascosporogenesis.

### Characterization of the ΔaptC Pigment and Noninvolvement of Putative Asperthecin Tailoring Genes

**Fig. 4:**
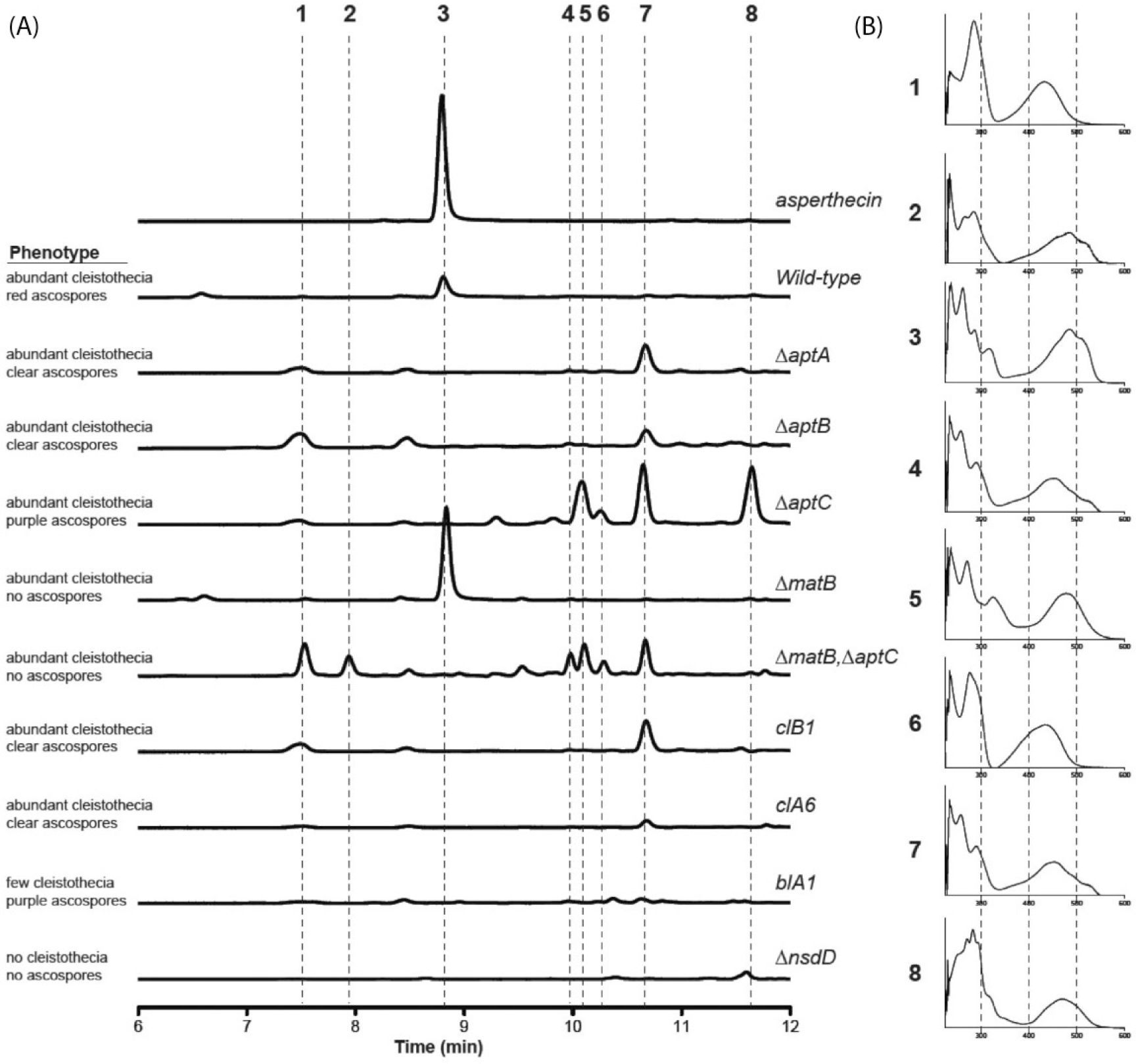
HPLC-DAD analysis of cleistothecial metabolites. Cleistothecia were collected, crushed, and extracted for metabolites from indicated strains of *Aspergillus nidulans*. (A) HPLC-PDA chromatograms of the crude extracts from each strain compared to the chromatogram of the standard asperthecin (3). Key peaks are highlighted by a dashed lines and details about acospore production of each mutant are provided on the left. (B) UV-Vis spectrum of the eight and compared to the UV-Vis spectrum of asperthecin (3) shown by comparison to a standard.

In order to learn more about the chemical profiles of the deletion mutants, we extracted metabolites under sexual development inducing conditions. HPLC-DAD analysis of metabolites extracted from crushed cleistothecia of the wild-type strain, various deletion mutants, and an asperthecin standard revealed the loss of asperthecin (Fig. 4: peak 3) in ΔaptA, ΔaptB, ΔaptC strains as well as in the corresponding mutants clA6, clB1, and blA1 (Fig. 4). We were surprised to see an accumulation of a new metabolite (Fig. 4: peak 7) that appeared in all deletion mutants except for blA1 (Fig. 4). Since the PKS (AptA) that synthesizes the carbon backbone of asperthecin is absent in the ΔaptA strain, peak 7 is therefore not derived from the Apt pathway. We also analyzed knockout strains of two genes involved in sexual development in *A. nidulans*, the positive acting GATA-type transcription factor encoding gene nsdD (Han et al., 2001) and the gene, MAT-1 (matB), encoding the “alpha-box” containing protein (Dyer et al., 2003), as well as a matB/aptC double deletion strain. Our data show an overproduction of asperthecin in ΔmatB and an absence of this compound in the ΔnsdD strain. Noteworthy is absence of the new metabolite (Fig. 4A: peak 7) in the ΔnsdD mutant. Analysis of ΔΔmatB/aptC mutant displayed almost the exact profile as the ΔaptC single deletion strain (Fig. 4).

As mentioned earlier, the original chemical characterization of the ascospore pigment resulted in structure elucidation of ascoquinone A (Brown & Salvo, 1994). However, this structure varies from asperthecin and a putative downstream metabolite of asperthecin, although no mass for ascoquinone A has been characterized experimentally (Brown & Salvo, 1994). We were most interested in trying to identify the structure of the purple pigment of the ΔaptC ascospores since the proposed functions of AptA, B, and C were biochemically elucidated in a heterologous expression host (Li et al., 2011). LCMS analysis of the extracts detected four metabolites, two prominent ones with respective m/z values of 355.0460 (5, C_18_H_11_O_8_), and 299.0196 (6, C_15_H_7_O_7_) in negative ionization mode (Supplementary Fig. S2); however, none of these metabolites correspond to the known intermediates described in the Apt pathway (Li et al., 2011). These data suggest that the metabolites detected are either shunt products of the Apt pathway, from different pathways, or represent an uncharacterized intermediate. Since asperthecin only contains the chemical makeup of a decarboxylated octaketide (C15), we speculated that during biosynthesis additional chain shortening had to occur.

Precedent for chain shortening reactions during pigment biosynthesis comes from *A. fumigatus* and *Botrytis cinerea*, where the hydrolase Ayg1 catalyzes such reactions (Fujii et al., 2004; Schumacher, 2016; Tsai et al., 2001). BlastP analysis of *A. fumigatus* Ayg1 querying the *A. nidulans* FGSC A4 genome resulted in one hit, AN9171 (63.7% identity, e-value: 4e-115), located on chromosome VI unlinked to the asperthecin gene cluster. As comparison, *A. fumigatus* Ayg1 and B cinerea Ayg1 share 52.4% identity (e-value: 5e-161). To determine if AygA was indeed involved in the hypothesized chain shortening reaction, we created ΔaygA and analyzed the secondary metabolite profile under sexually inducing conditions. However, the wild-type strain and the ΔaygA strains did not have any apparent difference and both still produced asperthecin (Supplementary Fig. S2). Therefore, the mechanism of chain shortening remains elusive.

## Discussion

In the past, secondary metabolites have been defined as metabolites that are not required for normal growth and development. However, many studies across fungal taxa show clear biological roles of secondary metabolites in fungal biology that are exceptions to this simplistic definition (Demain & Fang, 2000). For example, iron-binding siderophores are often defined as secondary metabolites; however, they play a key role in scavenging iron from environments, which is necessary for growth of many microbes (Haas et al., 2008). Xanthocillins, encoded by an isocyanide gene cluster, participate in copper homeostasis (Raffa et al., 2021). Some fungal secondary metabolites are required for asexual spore development (Zhang et al., 2017) and some even suppress fungal growth such as fusaristatin A produced by certain isolates of *Fusarium pseudograminearum* (Khudhair et al., 2020). Additionally, the literature is rich with examples of how fungal natural products provide protection/secure environmental niches when utilized as “weapons” against other microbes (Keller, 2019). Here we describe the “secondary” metabolite gene cluster responsible for production of the sexual spore pigment in *A. nidulans*, show it is required for normal ascosporogenesis, and protects ascospores from UV light.

The switch from vegetative growth to asexual or sexual development is a genetic regulatory pathway that is controlled by many factors in filamentous fungi. In a general sense, environmental stimuli such as changes in carbon, nitrogen, pH, and light are recognized by fungi which in term elicit a morphological change in growth. For the model fungus, *A. nidulans*, several stimuli are known to induce asexual or sexual development. Asexual development is induced by light through a genetic pathway involving a series of transcription factors (Yu, 2010) in which the end result is formation of conidiophores bearing specialized progenitor cells called phialides that produce conidia. Phialides are in essence the “stem cells” of asexual reproduction in *Aspergillus* as they repeatedly produce mitotically derived propagules. While the exact mechanisms are not understood, phialides also act as the “packaging center” for conidia where components of the cell wall are integrated, including conidial cell wall pigments. Whereas structural elucidation of the *A. nidulans* conidial pigment has remained elusive (postulated to be a complex polyketide polymer), the genes involved in production of the pigment are well known, a polyketide synthase *wA* (Mayorga & Timberlake, 1990) that synthesizes YWA1 intermediate which is then hypothesized to be polymerized by a laccase *yA* to form the green-colored pigment (O’Hara & Timberlake, 1989). Both of the enzymes, WA and YA, are specifically expressed in phialides but absent from conidia, indicating that the polymerized pigment is integrated into the conidial cell wall inside the phialide (Aramayo & Timberlake, 1993; O’Hara & Timberlake,1989).Numerous studies have reported the role of the conidial pigments for structural integrity of the conidial cell wall and protection from UV light in various fungi, particularly in the genus *Aspergillus* (Blachowicz et al., 2020; Geib et al., 2016; Wang et al., 2020; Zhang et al., 2017).

Sexual development is in turn induced by growth in the dark in which the coordinated activity of several transcription factors (Han, 2009) that govern formation of cleistothecia composed of ascogenous hyphae give rise to asci each containing eight ascospores. Consistent with the conidial pigment, we show that the sexual spore pigment is produced by the asperthecin PKS containing gene cluster (Fig. 2). Additionally, the asperthecin cluster is specifically expressed in cleistothecia (Fig. 1). Moreover, asperthecin protects ascospores from UV light (Fig. 3). In order to test if asperthecin specifically protects the ascospores from UV light-based DNA damage, we also tested the ascospores for sensitivity to a chemical mimic of UV light that induces the same DNA damage (pyrimidine-dimer formation). As expected, there was no differential sensitivity to MMS between the deletion mutants of the asperthecin gene cluster. These data indicate that the general DNA damage repair pathway is therefore not affected in asperthecin cluster deletion mutants; but rather, that asperthecin specifically protects ascospores from UV light. This is consistent with asperthecin absorbing light in the UV range (Fig.4B).The Δ*aptC* mutant metabolites also provide protection in the UV range, whereas, notably, there is an absence/diminution of any UV absorbing metabolites in the Δ*aptA* and Δ*aptB* mutants. We speculate that the enhanced resistance of Δ*aptC* ascospores to UV light, compared to wild type ascospores (Fig. 3), may be due to the numerous UV light absorbing metabolites made by this strain (Fig. 4B).

A consistent theme with spore pigments is that they form polymers which are thought to add structural rigidity. This is clearly documented in the fungus *Pestalotiopsis fici* where deletion of the conidial PKS results not only in loss of conidial pigmentation but also multicell conidial integrity (Zhang et al., 2017). Similarly, the polyketide-derived fusarubins contribute the dark colorization of the perithecial cell wall and their structural integrity (Studt et al., 2012). DHN melanin is an example of a spore pigment that is found in several filamentous fungi and is known to form large polymers (Ao et al., 2019; Pal et al., 2014; Schumacher, 2016). Due to the propensity for these pigments to polymerize, chemical characterization of spore metabolites is difficult. Brown and Salvo (1994) identified the sexual spore pigment of *A. nidulans* as ascoquinone A (MW = 619.0724); however, this identification was inferred by UV spectra and no mass corresponding to ascoquinone A was identified. We have repeatedly looked for a mass consistent with ascoquinone A and have also been unable to identify it via mass spectrometry of extracts. Instead, we were able to consistently identify asperthecin (MW = 318.0376) by HPLC and mass spectrometry. Interestingly, ascoquinone A is composed of two asperthecin molecules joined with an ester linkage. While we were unable to generate evidence to definitively support the claim that asperthecin polymerizes as a structural component of the ascospore cell wall—given the polymerization theme in other spore pigments, the requirement of asperthecin for normal ascospore development, and asperthecin being a monomer of ascoquinone A—we think it possible that asperthecin forms polymers as an integral part of the ascospore cell wall that has a specific function in ascospore cell wall maturation and protection from UV light.

Surprisingly, the Δ*aptC* mutant accumulated several unique masses although none were the known intermediates of the Apt pathway reconstituted in *Saccharomyces cerevisiae* (Li et al., 2011). Finally, we were able to assign two historical clear ascospore mutants (*clA6* and *clB1*) to specific mutations in the *aptA* and *aptB* genes, respectively. What remains a mystery is the explanation of the *blA1* mutant, which despite producing ascospores that superficially resemble the Δ*aptC* ascospores, we were unable to find any mutations in *aptC*.

## Supplementary Material

supplementary

## Figures and Tables

**Table 1:** *Aspergillus nidulans* Strains Used in this Study

Strain	Genotype	Source

RJMP103.5	Wild type	(Palmer et al., 2013)
LO2131	*pyroA4, pyrG89, riboB2,* Δ*aptA::pyroA,* Δ*sumO::pyrG,* Δ*nkuA, veA1*	(Szewczyk et al., 2008)
LO2435	*pyroA4, pyrG89, riboB2,* Δ*aptB::pyroA,* Δ*sumO::pyrG,* Δ*nkuA, veA1*	(Szewczyk et al., 2008)
LO2440	*pyroA4, pyrG89, riboB2,* Δ*aptC::pyroA,* Δ*sumO::pyrG,* Δ*nkuA, veA1*	(Szewczyk et al., 2008)
RDIT55.37	*pyroA4, veA+*	(Tsitsigiannis et al., 2005)
RJMP240.8	Δ*aptA, veA+*	This study
RJMP238.5	Δ*aptB, veA+*	This study
RJMP239.7	Δ*aptC, veA+*	This study
TJMP190.4	*pyrG89, pyroA4,* Δ*matB::pyrG,* Δ*nkuA, veA+*	This study
RJMP290.2	Δ*matB, veA+*	This study
RJMP291.12	Δ*matB,* Δ*aptC,* Δ*nkuA, veA+*	This study
RJMP139.13	*pyroA4, pyrG89, metG1, veA+*	This study
FGSCA674	*clB1, yA2, wA2, sC12*	FGSC
FGSCA280	*clA6, proA1, pabaA1, yA2, palB7*	FGSC
FGSCA268	*blA1, yA2, wA3, thiA4, cxnE16, adeD3*	FGSC
RJMP250.1	*pyroA4, veA+*	This study
RJMP251.3	*clA6, pyroA4, veA+*	This study
RJMP252.14	*clB1, pyroA4, veA+*	This study
RJMP250.11	*blA1, pyroA4, veA+*	This study
RDIT88.13	Δ*nsdD, veA+*	(Tsitsigiannis et al., 2004)
RJMP1.49	*pyroA4, pyrG89,* Δ*nkuA, veA+*	(Shaaban et al., 2010)
RCG1.4	*pyroA4, pyrG89, riboB2,* Δ*aptC::pyroA,* Δ*sumO::pyrG,* Δ*nkuA, veA+*	This study
TCG1.1	*pyroA4, pyrG89, riboB2,* Δ*aptC::pyroA,* Δ*aygA::riboB,* Δ*sumO::pyrG,* Δ*nkuA, veA+*	This study
TCG9.1	*pyroA4,* Δ*nkuA::argB,* Δ*aygA::pyroA, veA+*	This study

**Table 2:** Genes Deleted in *blA1* Mutant

Gene name	Putative function

AN3482	hypothetical, putative end of chromosome
AN3483	hypothetical
AN3484	hypothetical
AN3485	hypothetical
AN3486	hypothetical
AN3487	putative glutathione S transferase
AN3488	putative cyclohexanone monooxygenase
AN3489	putative ER transporter homology
**AN3490**	**InpC**
**AN3491**	**InpD**
**AN3492**	**ScpR**
**AN3493**	**InpE**
**AN3494**	**InpF**
**AN3495**	**InpA**
**AN3496**	**InpB**
AN3497	putative p450 monooxygenase
AN3498	putative MFS transporter
AN3499	putative anhydro-N-acetylmuramic acid kinase
AN3500	putative retropepsin

*Note.* Fellutamide biosynthetic gene cluster shown in bold.

## Data Availability

Raw sequencing data is available via the NCBI Small Read Archive under the SRP326812 accession and the corresponding BioProject PRJNA743544 accession.
