# Supplementary material for: The sexual spore pigment asperthecin is required for normal ascospore production and protection from UV light in Aspergillus nidulans
Source: J Ind Microbiol Biotechnol. 2021 Aug 20;48(9-10):kuab055. doi: 10.1093/jimb/kuab055 (PMC8762651; doi:10.1093/jimb/kuab055)
Supplement: kuab055_Supplemental_File [file kuab055_supplemental_file.docx]

**Supporting Information**

**The sexual spore pigment asperthecin is required for normal ascospore production and protection from UV light in *Aspergillus nidulans*.**

Jonathan M. Palmer^1,4^, Philipp Wiemann^1^, Claudio Greco^1^, Yi Ming Chiang^2^, Clay C.C. Wang^2^, Daniel L. Lindner^4^, and Nancy P. Keller^1,3,^ *

^1^ Department of Medical Microbiology & Immunology, University of Wisconsin-Madison, Madison, WI 53706

^2^ Departments of Chemistry and Pharmacology & Pharmaceutical Sciences, University of Southern California, Los Angeles, CA 90089

^3^ Department of Bacteriology, University of Wisconsin-Madison, Madison, WI 53706

^4^ Center for Forest Mycology Research, Northern Research Station, US Forest Service, Madison, WI 53726

* Corresponding Author:

Nancy P. Keller

3467 Microbial Sciences

1550 Linden Drive

Madison, WI 53706

Email: [npkeller@wisc.edu](mailto:npkeller@wisc.edu)

Phone: (608) 262-9795

**Figure S1.** Southern results for ∆*aygA*. A Schematic of the expected product from digestion using EcoRI and EcoRV for the ∆*aygA* mutant and wildtype. B Southern results for ∆*aygA*.

**
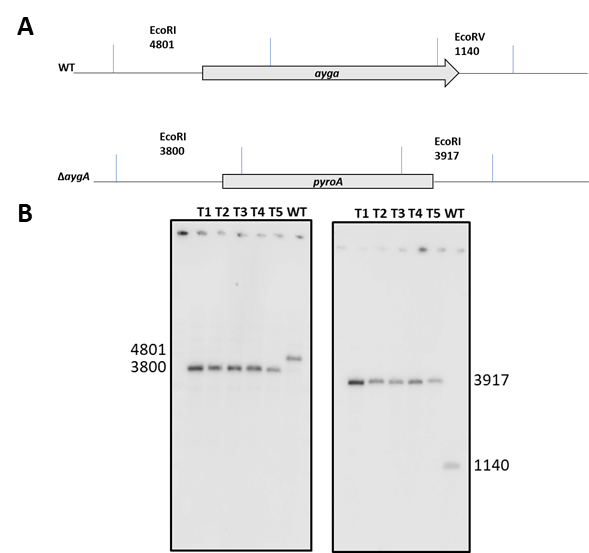
**

**Figure S2.** Analysis of crude extracts from cleistothecia by UHPLC-HRMS; A) total ion chromatograms in negative mode for the WT, ∆*aygA* and ∆*aptC* strains. B) Extracted ion chromatogram for 317 (asperthecin), showing that asperthecin is only produced in the WT and ∆*aygA* but not in the ∆ mutant. Peaks labelled **3** corresponds to asperthecin (317.0303 [M-H]^-^) and **5** and **6** to putative metabolites with masses of 355.0462 [M-H]^-^) and 299.0200 [M-H]^-^ respectively.

**
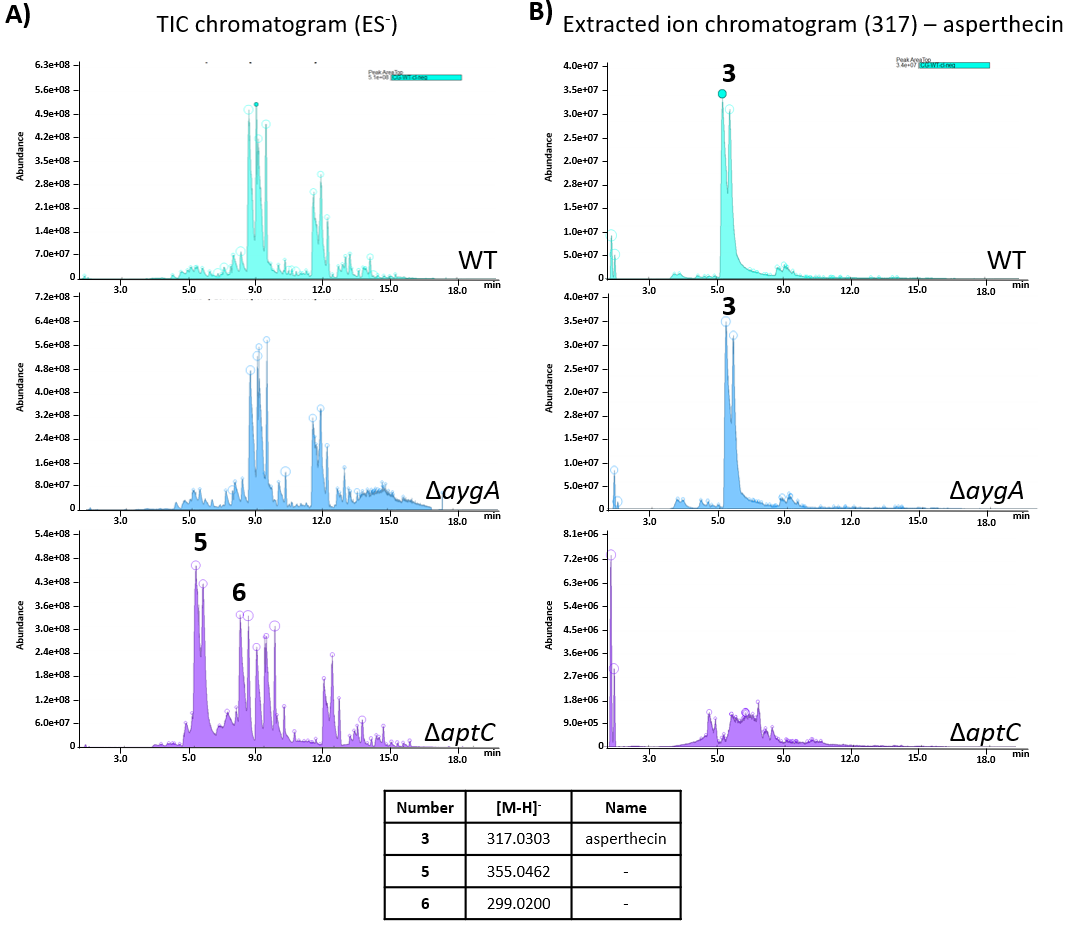
**

Table S1. List of oligonucleotides

| **Name** | **Sequence** | **Use** |
| --- | --- | --- |
| CG-RiboB-F | TGAATCAAGGCGGACTGA | Selectable marker |
| CG-RiboB-R | TGCCACTCAACGCCATT | Selectable marker |
| CG-pyrG-F | TGCTCTTCACCCTCTTCGC | Selectable marker |
| CG-pyrG-R | CTGTCTGAGAGGAGGCACTG | Selectable marker |
| CG-PtrA-F | CAATTGATTACGGGATCCCATTGGTAACG | Selectable marker |
| CG-PtrA-R | CTCTTGCATCTTTGTTTGTATTATACTGTC | Selectable marker |
| CG-Ayg1-LF | AACGACTAGTAAGCGGATTGG | Gene deletion |
| CG-Ayg1-LR | GTCATCCATAACTCAGTCCGCCTTGATTCACGTGTTCCTGTATATGAGAC | Gene deletion |
| CG-Ayg1-RF | AGATCACTGAGTCAATGGCGTTGAGTGGCAGTTAGACTGGCAGGTAGCAA | Gene deletion |
| CG-Ayg1-RR | AAGTAGTCGAGGCAAGTAGC | Gene deletion |
| CG-Ayg1-LF-int | GTTTGCAGCAAGTCCTCCA | Gene deletion |
| CG-Ayg1-RR-int | GCTAGCGACGTGGTATACTCA | Gene deletion |
| CG-pyrG-Ayg1-LR | ATTTCAGACCCGCGAAGAGGGTGAAGAGCACGTGTTCCTGTATATGAGAC | Gene deletion |
| CG-pyrG-Ayg1-RF | CATCACGCATCAGTGCCTCCTCTCAGACAGGTTAGACTGGCAGGTAGCAA | Gene deletion |
| CG-pyrG-LccB-LR | ATTTCAGACCCGCGAAGAGGGTGAAGAGCAGTTCACAGAACGCTGATTG | Gene deletion |
| CG-ptra-Ayg1-LR | CGTTACCAATGGGATCCCGTAATCAATTGCGTGTTCCTGTATATGAGAC | Gene deletion |
| CG-ptrA-Ayg1-RF | GACAGTATAATACAAACAAAGATGCAAGAGGTTAGACTGGCAGGTAGCAA | Gene deletion |
| CG-ayg1KOribo-check-F | GGGAGAAGTACCATGCGGAATC | Check deletion |
| CG-Ayg1-Check-LF | GCGGAATCCGTCTACCATCA | Check deletion |
| CG-prtA-Check-LR | CCAACAGCTTGGTCTGAGAAG | Check deletion |
| CG-Ayg1-Check-RR | GGAGGAGCTGGTTTTGGATC | Check deletion |
| CG-prtA-Check-RF | CGACACTCATGTCGAAGGTTC | Check deletion |
| CG-ayg1-purity-LR | CGCATTCATGAAATCGTCAGCC | Check deletion |
